# Be a Mom: Patterns of Program Usage and Acceptability Among Women With Low-Risk and High-Risk for Postpartum Depression

**DOI:** 10.3389/fgwh.2022.841427

**Published:** 2022-03-17

**Authors:** Sandra Xavier, Fabiana Monteiro, Maria Cristina Canavarro, Ana Fonseca

**Affiliations:** Faculty of Psychology and Educational Sciences, Center for Research in Neuropsychology and Cognitive Behavioral Intervention, University of Coimbra, Coimbra, Portugal

**Keywords:** web-based intervention, Be a Mom, feasibility, acceptability, postpartum depression risk

## Abstract

**Background:**

Be a Mom is a self-guided web-based intervention developed to prevent postpartum depression (PPD) symptoms and to promote maternal wellbeing, respectively among high and low-risk new mothers. This study aims to examine and compare (1) Be a Mom's patterns of usage and (2) Be a Mom's acceptability among women presenting high and low risk for PPD.

**Methods:**

The sample was composed by 800 women who were randomized to Be a Mom [542 presenting high-risk (Postpartum Depression Predictors Inventory-Revised ≥ 5.5) and 258 presenting low-risk for PPD]. Data regarding patterns of usage were collected through the Be a Mom website. Acceptability data were collected through a brief questionnaire.

**Results:**

27.9% of high-risk and 36.3% of low-risk women completed the program. A higher proportion of participants in the low-risk group completed Be a Mom [$X_{\left(1\right)}^{2}$ = 5.29, *p* = 0.021] and completed more modules [*t*_(723)_ = −3.01, *p* = 0.003]. No significant differences were found between the groups in number of logins, minutes spent on the program, exercises completed and number of times audios were played. a higher proportion of women in the high-risk group considered that participating in Be a Mom was too demanding [$X_{\left(1\right)}^{2}$ = 8.21, *p* = 0.004].

**Conclusions:**

Despite the low rates of completion, Be a Mom appears to be an acceptable option for both women with high-risk and low-risk for PPD. Lack of time seems to be the main reason for non-completion, so it is important to develop briefer versions of the program and introduce engagement strategies that may increase completion rate.

## Introduction

The postpartum period represents a demanding and vulnerable period where women are at increased risk for mental health disorders (1, 2). Postpartum depression (PPD) is one of the most prevalent mental health problems in the postpartum period and affects ~10–15% of mothers (3, 4). There is a general consensus that PPD has adverse consequences for mothers, infants, and their families (5, 6). Although during pregnancy or in the early postpartum it is possible to identify women with higher risk to develop PPD (e.g., with a range of demographic, social, and psychological risk factors) (7, 8), studies have shown that low-risk women may also experience symptoms of depression, anxiety and insomnia in the postpartum period (9).

Research studies have demonstrated that Cognitive Behavioral Therapy (CBT) for PPD effectively reduced levels of depressive symptoms and decreased the risk of PPD (10, 11). Literature has also suggested that preventive interventions for PPD were similarly effective in women with clinical symptoms of depression, in women who presented risk factors for PPD and in women in the community (10).

Additionally, evidence has showed that not only prevention of mental illness should be targeted, but also the promotion of positive mental health should be incorporated in preventive and treatment intervention programs in the postpartum period (12). So, it seems that both women with high and low-risk for PPD could benefit from these intervention programs to reduce depression symptoms and promote their mental health, regardless of their risk level for developing PPD.

Nonetheless, several postpartum women face substantial barriers to engage in conventional interventions to prevent depressive symptoms (e.g., lack of recognition of PPD signs and symptoms, lack of time, stigma, financial and childcare issues, etc.) (13–15) and the majority of them do not actively seek help for their mental health problems (16, 17). In order to alleviate these barriers, to reach a greater number of postpartum women, to provide accessible evidence-based help and support to prevent PPD and improve cost-effectiveness, web-based psychological interventions can be assumed as a promising approach that may be particularly engaging and useful (1, 14, 16, 18). In fact, web-based interventions were found to be an effective solution for reducing anxiety and depressive symptoms (19–21) and improving psychological wellbeing and positive mental health (22, 23) among postpartum women, and have shown to be feasible and acceptable in perinatal samples (18, 24–26).

According to this, Be a Mom, a short-term self-guided web-based intervention, grounded in CBT principles and integrating third-wave CBT approaches (e.g., self-compassion and acceptance and commitment therapy), was developed to prevent PPD symptoms and to promote maternal wellbeing among postpartum women. Be a Mom was originally developed to prevent PPD among at-risk women (27) and afterwards it was applied among low-risk women (23). Results have demonstrated evidence of Be a Mom's efficacy in reducing depressive and anxiety symptoms (27) and promoting self-compassion and emotion regulation skills (21) among a sample of women with high-risk for PPD, as well as of Be a Mom's efficacy in increasing positive mental health among a sample of women with low-risk for PPD (23). Moreover, Be a Mom was found to be an acceptable intervention for both women with high-risk (27) and low-risk (23) for PPD. Nonetheless, Be a Mom's feasibility results have showed a low adherence to the intervention (23, 27). As previous studies illustrate, dropout rates of web-based interventions in the postpartum period tend to be high (22, 23, 27, 28). However, there is still much to explore and further studies are needed to understand the patterns of usage and acceptability of these interventions among postpartum women, and also to understand similarities and differences among high-risk and low-risk women. Therefore, the aim of this study was to explore Be a mom's patterns of usage, reasons for dropout and acceptability and access to the program among women presenting high and low-risk for PPD.

## Methods

### Procedure

This study consists of a secondary analysis of a wider research project assessing Be a Mom's efficacy as a web-based program for the promotion of maternal mental health and prevention of depression during the postpartum period. The study was approved by the Ethics Committee of the Faculty of Psychology and Educational Sciences, University of Coimbra.

Participants' enrolment in the study occurred between January 2019 and February 2021. The inclusion criteria were as follows: (i) being 18 years or older, (ii) being in the early postpartum period (up to 3 months postpartum), (iii) having a computer/tablet/smartphone and internet access at home; (iv) having the ability to read and speak Portuguese; and (v) being a Portugal resident. Exclusion criteria were the presence of a serious medical condition (physical or psychiatric) in the mother or in the infant (self-reported).

Recruitment occurred through online advertisements (unpaid cross-posting, paid advertisements and boosting campaigns) on social media networks (e.g., Facebook and Instagram) and on websites focusing on maternity themes. It was used a secure web form (LimeSurvey®) which presented information about the study goals, procedures, the voluntary nature of the participation, the participant's right to withdraw the study at any time and the ethical considerations regarding anonymity and confidentiality.

Only those who gave their informed consent (by answering affirmatively to the question “I understand and accept the conditions of the study”) and agreed to the study's conditions were screened for eligibility and asked to provide their contact information. The participants who met the eligibility criteria were divided in high-risk and low-risk groups, considering their scores in the Postpartum Depression Predictors Inventory-Revised (PDPI-R) (high-risk for PPD, PDPI-R ≥ 5.5). High-risk and low-risk participants were sent an email with a link to complete the baseline assessment (Time 1—T1). After completing the baseline assessment, participants in high and low-risk groups were randomly assigned to the intervention group (Be a Mom) or to the waiting-list control group with a 1:1 allocation through a computer-generated random number.

Women in the intervention groups were invited to access the Be a Mom program (beamom.pt), which is composed of 5 sequential modules (Changes and Emotional Reactions; Cognitions; Values and Social Support; Couple's Relationship and PPD Alert Signs and Professional Help-seeking). The program is completely self-guided, with asynchronous communication channels for technical support, and did not include peer support forum during the use of the program. Eight weeks after randomization participants were asked to fill the post-intervention assessment (Time 2—T2). Data regarding patterns of usage were retrieved from Be a Mom's website system data. In the current study, only data from the women allocated to the intervention group (both from the high-risk and low-risk groups) were considered.

### Measures

#### Sociodemographic and Clinical Information

Participants' sociodemographic (e.g., age, marital status, number of children, socioeconomic status, employment status, educational level, average monthly income, and residence), clinical (psychopathology history, psychological/psychiatric treatment history) and infant (e.g., age, gender and infant's gestational age at birth) information were collected through a self-report form.

#### Be a Mom's Patterns of Usage

Data were collected in Be a Mom's website system concerning number of logins, average minutes spent on the program, number of completed modules and finished exercises, and number of times each audio exercise was played.

#### Reasons for Dropout

The participants who did not complete the program (completed at least the first three modules) were asked to select one or more reasons for dropping out of the program.

#### Be a Mom's Acceptability and Access

Participants were asked about the meaning, understanding and usefulness of strategies/activities on everyday life for each module using a dichotomous scale (Yes/No). Regarding overall satisfaction with the program, participants were asked about their satisfaction with the help provided, intentions to use it again if needed, if they would recommend it to a friend, the usefulness/relevance of the information and burden (time-commitment required). All questions relating to satisfaction were answered on a dichotomous response scale (0 = Not applicable to me and 1 = Applicable to me). The quality of the program was assessed through a 4-point Likert scale (from 0 = Very bad to 4 = Excellent). Participants were also asked about the presence/participation of others when accessing the modules (“Has anyone accessed the Be a Mom modules with you?”, and “If yes, who?”).

### Data Analysis

Statistical analyses were conducted using the Statistical Package for the Social Sciences (IBM SPSS, version 25.0). Descriptive statistics were performed to describe the sociodemographic and clinical characteristics, Be a Mom's patterns of usage, acceptability and access of the sample. Comparison tests (*t*-tests and chi-squared tests) were used to explore and compare these variables among high-risk and low-risk intervention groups. No control was made for multiplicity of tests. *P-*values presented are purely nominal (i.e., for each comparison).

## Results

### Participants

The total sample comprised 800 postpartum women that were randomized and allocated to the intervention groups, of which 542 presented high-risk for PPD and 258 low-risk for PPD. Sociodemographic and clinical characteristics of the total sample and high-risk and low-risk groups in the baseline assessment (T1) are presented in Table 1. The participants' mean age was 32.94 years old (*SD* = 4.25), most women were currently married/co-habiting (*n* = 731, 91.4%), and 66.5% (*n* = 532) were primiparous. The majority of women was currently employed (*n* = 700, 87.6%) and had a Master's or Doctorate degree (*n* = 293, 36.6%). Concerning the clinical characteristics, 37.6% (*n* = 301) of women had psychiatric/psychological problems history, and 37.8% (*n* = 302) reported previous psychiatric/psychological treatment and 9.4% (*n* = 75) reported currently having psychiatric/psychological treatment.

**Table 1:** Participants' sociodemographic and clinical characteristics at baseline.

	**Total sample (*n* = 800)**	**High-risk intervention group (*n* = 542)**	**Low-risk intervention group (*n* = 258)**	**t*/χ*^2^**
**Sociodemographic characteristics**				
**Age (years)**, ***M*** **(SD)**	32.94 (4.25)	32.91 (4.40)	33.02 (3.92)	−0.35
**Marital status**, ***n*** **(%)**				13.77^**^
Married/co-habiting	731 (91.4)	482 (88.9)	249 (96.5)	
Single	33 (4.1)	28 (5.2)	5 (1.9)	
Divorced or separated	11 (1.4)	11 (2.0)	-	
In a relationship (without living together)	25 (3.1)	21 (3.9)	4 (1.6)	
**Primiparous**, ***n*** **(%)**	532 (66.5)	347 (64.0)	185 (71.7)	1.75
**Educational level**, ***n*** **(%)**				21.94^***^
Basic (up to the 9th grade)	24 (3.0)	22 (4.1)	2 (0.8)	
Secondary (10th−12th grade)	165 (20.6)	131 (24.2)	34 (13.2)	
Bachelor's degree	69 (8.6)	44 (8.1)	25 (9.7)	
Master's or Doctorate	293 (36.6)	181 (33.4)	112 (43.4)	
Other or did not answer	249 (31.1)	164 (30.3)	85 (32.9)	
**Professional status**, ***n*** **(%)**				4.84
Employed	700 (87.6)	465 (85.8)	235 (91.1)	
Unemployed	72 (9.0)	57 (10.5)	15 (5.8)	
Student or housewive	28 (3.5)	20 (3.7)	6 (2.3)	
**Household monthly income**, ***n*** **(%)**				26.09^***^
<580€	78 (9.8)	68 (12.5)	10 (3.9)	
580€−1,000€	390 (48.8)	276 (50.9)	114 (44.2)	
1,000€−2,000€	294 (36.8)	173 (31.9)	121 (46.9)	
2,000€−3,500€	36 (4.5)	24 (4.4)	12 (4.7)	
More than 3,500€	2 (0.3)	1 (0.2)	1 (0.4)	
**Residence**, ***n*** **(%)**				4.21^*^
Urban	624 (78.0)	434 (80.1)	190 (73.6)	
Rural	176 (22.0)	108 (19.9)	68 (26.4)	
**Clinical characteristics**				
**Psychiatric/psychological problems history** ***n*** **(%)**				31.72^***^
Yes	301 (37.6)	240 (44.3)	61 (23.6)	
No	499 (62.4)	302 (55.7)	197 (76.4)	
**Previous psychiatric/psychological treatment**, ***n*** **(%)**				18.27^***^
Yes	302 (37.8)	232 (42.8)	70 (27.1)	
No	498 (62.3)	310 (57.2)	188 (72.9)	
**Current psychiatric/psychological treatment**, ***n*** **(%)**				4.60
Yes	75 (9.4)	59 (11.0)	16 (6.3)	
No	725 (90.6)	483 (89.1)	242 (93.8)	
**Infant's characteristics**				
**Age (in months)**, ***M*** **(SD)**	1.98(0.98)	2.06 (0.98)	1.80 (0.95)	3.59^**^
**Gestational weeks (at birth)**, ***M*** **(SD)**	38.75 (3.06)	38.66 (3.55)	38.95 (1.60)	−1.26
**Sex**, ***n*** **(%)**				0.70
Male	391 (48.9)	260 (48.0)	131 (50.8)	
Female	398 (49.8)	275 (50.7)	123 (47.7)	

* *p < 0.05*.

** *p < 0.01*.

*** *p < 0.001*.

*Bold values represent significant differences between groups*.

There were significant differences between high-risk and low-risk intervention groups in some sociodemographic and clinical characteristics. Regarding marital status, a higher proportion of women in the low-risk intervention group were currently married/co-habiting compared to the high-risk group [$X_{\left(3\right)}^{2}$ = 13.77, *p* = 0.003]. Considering education level, women in the high-risk intervention group had lower education levels than women in the low-risk group [$X_{\left(3\right)}^{2}$ = 21.94, *p* < 0.001]. Regarding household monthly income, a higher proportion of participants in the high-risk intervention group had an income lower than 580€ and a higher proportion of participants in the low-risk intervention group had an income of 1,000–2,000€ [$X_{\left(4\right)}^{2}$ = 26.09, *p* < 0.001]. Moreover, a higher proportion of women in the high-risk group lived in an urban area [$X_{\left(1\right)}^{2}$ = 4.21, *p* = 0.040] and had an older infant [*M* = 2.06 months*, SD* = 0.98 vs. *M* = 1.80 months*, SD* = 0.95, *t*_(798)_ = 3.59, *p* < 0.001] compared to the low-risk group. Concerning the clinical characteristics, when compared to the low-risk group, a higher proportion of women in the high-risk group had psychiatric/psychological problems history [$X_{\left(1\right)}^{2}$ = 31.72, *p* < 0.001] or previous or current psychiatric/psychological treatment [$X_{\left(1\right)}^{2}$ = 18.27, *p* < 0.001].

Of the 800 women invited to access Be a Mom's website, 725 (90.6%) registered on the website. Significant differences in some sociodemographic characteristics between women who registered and women who did not register on Be a Mom's platform were found. Women who did not register were older [*M* = 33.88 years, *SD* = 4.27 vs. *M* = 32.85 years*, SD* = 4.24, *t*_(798)_ = −2.01, *p* < 0.05], had a higher number of children [*M* = 1.61 *SD* = 0.70 vs. *M* = 1.35*, SD* = 0.58, *t*_(798)_ = −3.13, *p* = 0.002] and had lower education levels [$X_{\left(3\right)}^{2}$ = 8.40, *p* = 0.038] compared to women who registered on Be a Mom's website.

### Be a Mom's Patterns of Usage

Of the 725 (90.6% of total sample) who registered at the platform, 491 (67.7%) belonged to the high-risk and 234 (32.3%) to the low-risk group. Table 2 presents the results regarding Be a Mom's patterns of usage (number of logins, average minutes spent on the program, mean of completed modules, finished exercises and audio exercises) in total sample and intervention groups. In total, 222 (30.6%) participants completed the Be a Mom program (at least 80% −4 out of 5 modules completed) and 155 (21.4%) participants were partial completers of the program (completed at least 2 or 3 modules). Participants had an average number of logins of 5.41 (*SD* = 4.72) and spent an average of 14.87 (*SD* = 11.06) minutes on the program's website.

**Table 2:** Participants' patterns of program usage.

	**Total sample (*n* = 725)**	**High-risk intervention group (*n* = 491)**	**Low-risk intervention group (*n* = 234)**	***t*/χ^2^**
**Completed Be a Mom**, ***n*** **(%)**				5.29^*^
Yes	222 (30.6)	137 (27.9)	85 (36.3)	
Partial completers	155 (21.4)	102 (20.8)	53 (22.6)	
No	348 (48.0)	252 (51.3)	96 (41.1)	
**Modules completed**, ***M*** **(SD)**	2.21 (1.98)	2.06 (1.96)	2.53 (1.98)	−3.01^**^
**Number of logins**, ***M*** **(SD)**	5.41 (4.72)	5.19 (4.52)	5.88 (5.08)	**–**1.84
**Minutes spent on program**, ***M*** **(SD)**	14.83 (11.06)	14.31 (10.95)	15.91 (11.25)	**–**1.81
**Finished exercises in module 1**, ***M*** **(SD)**	3.31 (1.23)	3.26 (1.26)	3.41 (1.17)	**–**1.53
**Finished exercises in module 2**, ***M*** **(SD)**	1.52 (0.76)	1.51 (0.74)	1.52 (0.79)	−0.135
**Finished exercises in module 3**, ***M*** **(SD)**	2.98 (1.53)	2.93 (1.53)	3.05 (1.52)	−0.721
**Finished exercises in module 4**, ***M*** **(SD)**	1.58 (0.79)	1.57 (0.80)	1.59 (0.79)	−0.260
**Finished exercises in module 5**, ***M*** **(SD)**	1.39 (0.65)	1.39 (0.67)	1.38 (0.64)	0.195
**Audio exercises**, ***M*** **(SD)**				
Audio 1 (thoughts supression)	0.52 (0.85)	0.48 (0.70)	0.57 (1.11)	−1.24
Audio 2 (distancing from thoughts)	0.32 (0.76)	0.29 (0.55)	0.40 (1.08)	−1.44

* *p < 0.05*.

** *p < 0.01*.

*Bold values represent significant differences between groups*.

As can be seen in Table 2, when compared with the high-risk group, a higher proportion of participants in the low-risk group completed Be a Mom [$X_{\left(1\right)}^{2}$ = 5.29, *p* = 0.021], and completed more modules [*t*_(723)_ = −3.01, *p* = 0.003]. No significant differences were found between the high and low-risk intervention groups in the average number of logins [*t*_(723)_ = −1.84, *p* = 0.066], minutes spent on the program [*t*_(723)_ = −1.81, *p* = 0.070], exercises completed in each module [Module 1: *t*_(723)_ = −1.53, *p* = 0.128; Module 2: *t*_(531)_ = −0.135, *p* = 0.893; Module 3: *t*_(375)_ = −0.721, *p* = 0.471; Module 4: *t*_(268)_ = −0.260, *p* = 0.795 and Module 5: *t*_(220)_ = −0.195, *p* = 0.845] and number of times audio 1 [*t*_(723)_ = −1.24, *p* = 0.217] and audio 2 [*t*_(723)_ = −1.44, *p* = 0.151] were played.

### Reasons for Not Completing Be a Mom

Table 3 presents the results concerning the reasons for not completing Be a Mom.

**Table 3:** Participants' reasons for not completing Be a Mom.

	**Total sample (*n* = 211)**	**High-risk intervention group (*n* = 134)**	**Low-risk intervention group (*n* = 77)**	**χ^2^**
**Reasons for dropout**, ***n*****, %**				
Personal reasons (e.g., disease)	20 (9.5)	14 (10.4)	6 (7.8)	0.402
Not confortable with an online format	6 (2.8)	5 (3.7)	1 (1.3)	1.05
Did not like the contents of the program	1 (0.5)	1 (0.7)	0 (0.0)	0.577
Not useful	12 (5.7)	9 (6.7)	3 (3.9)	0.725
Lack of time	192 (91.0)	125 (93.3)	67 (87.0)	2.35
Technical problems	19 (9.0)	12 (9.0)	7 (9.1)	0.001
Difficulty in usage	2 (0.4)	2 (1.5)	0 (0.0)	1.16
Internet format is not ideal to approach the contents of the program	3 (1.4)	2 (1.5)	1 (1.3)	0.013
Did not complete the program but intended to complete	9 (4.3)	4 (3.0)	5 (6.5)	1.47
Program too extensive/too much information	4 (1.9)	2 (1.5)	2 (2.6)	0.321
Forgot the program's time limit	1 (0.5)	0 (0.0)	1 (1.3)	1.75

Of the 503 participants who did not complete Be a Mom, 211 answered a questionnaire about their reasons for not completing the program. The most frequently indicated reason for not completing the program was lack of time (*n* = 192, 91%), followed by personal issues (*n* = 20, 9.5%), technical problems (*n* = 19, 9%), feeling that the program was not useful (*n* = 12, 5.7%) or not feeling comfortable with the online format (*n* = 6, 2.8%). Moreover, 9 women (4.3%) reported that they did not complete Be a Mom but intended to complete. No significant differences in the reasons for not completing Be a Mom were found as a function of group (high-risk vs. low-risk women).

### Be a Mom's Acceptability and Access

Table 4 presents data concerning the acceptability of Be a Mom.

**Table 4:** Be a Mom's acceptability.

	**Total sample**	**High-risk intervention group**	**Low-risk intervention group**	**χ^2^**
**Modules' satisfaction**, ***n*****, %**				
**Module 1**				
**Meaningful information**				0.534
Yes	531 (99.8)	346 (99.7)	185 (100.0)	
No	1 (0.2)	1 (0.3)	-	
**Understanding the information**				1.64
Yes	526 (99.4)	340 (99.1)	186 (100.0)	
No	3 (0.6)	3 (0.9)	-	
**Usefulness of strategies/activities on everyday life**				2.00
Yes	512 (96.2)	331 (95.4)	181 (97.8)	
No	20 (3.8)	16 (4.6)	4 (2.2)	
**Module 2**
**Meaningful information**				0.023
Yes	363 (98.4)	231 (98.3)	132 (98.5)	
No	6 (1.6)	4 (1.7)	2 (1.5)	
**Understanding the information**				0.029
Yes	366 (98.7)	233 (98.7)	133 (98.5)	
No	5 (1.3)	3 (1.3)	2 (1.5)	
**Usefulness of strategies/activities on everyday life**				0.007
Yes	354 (95.7)	225 (95.7)	129 (95.6)	
No	16 (4.3)	10 (4.3)	6 (4.4)	
**Module 3**				
**Meaningful information**				
Yes	257 (98.1)	160 (98.2)	97 (98.0)	0.011
No	5 (1.9)	3 (1.8)	2 (2.0)	
**Understanding the information**				0.289
Yes	257 (98.5)	159 (98.1)	98 (99.0)	
No	4 (1.5)	3 (1.9)	1 (1.0)	
**Usefulness of strategies/activities on everyday life**				0.019
Yes	251 (96.2)	156 (96.3)	95 (96.0)	
No	10 (3.8)	6 (3.7)	4 (4.0)	
**Module 4**				
**Meaningful information**				3.09
Yes	213 (97.7)	131 (96.3)	82 (100.0)	
No	5 (2.3)	5 (3.7)	-	
**Understanding the information**				**-**
Yes	217 (100.0)	135 (100.0)	82 (100.0)	
No	-	-	-	
**Usefulness of strategies/activities on everyday life**				1.20
Yes	215 (99.1)	134 (98.5)	81 (100.0)	
No	2 (0.9)	2 (1.5)	-	
**Overall satisfaction with Be a Mom**, ***n*****, %**	***n*** **=** **427**	***n*** **=** **267**	***n*** **=** **160**	
Satisfation with the help provided	305 (71.4)	196 (73.4)	109 (68.1)	1.37
Recommend to a friend	363 (85.0)	228 (85.4)	135 (84.4)	0.081
Use it again if needed	328 (76.8)	211 (79.0)	117 (73.1)	1.96
Relevant information learned	325 (76.1)	207 (77.5)	118 (73.8)	0.785
Too demanding	105 (24.6)	78 (29.2)	27 (16.9)	8.21^**^
Participation was not worth it	45 (10.5)	30 (11.2)	15 (9.4)	0.367

** *p < 0.01*.

*Bold values represent significant differences between groups*.

Regarding the modules' satisfaction, the majority of participants considered the information meaningful (Module 1: *n* = 531, 99.8%; Module 2: *n* = 363, 98.4%; Module 3: *n* = 257, 98.1% and Module 4: *n* = 213, 97.7%), understandable (Module 1: *n* = 526, 99.4%; Module 2: *n* = 366, 98.7%; Module 3: *n* = 257, 98.5% and Module 4: *n* = 217, 100%), and reported that the strategies/activities suggested in each module were useful in everyday life (Module 1: *n* = 512, 96.2%; Module 2: *n* = 354, 95.7%; Module 3: *n* = 251, 96.2% and Module 4: *n* = 215, 99.1%). When comparing high and low-risk groups, no significant relationship was found between the groups and modules' satisfaction (i.e., meaningfulness and understanding of the information, and usefulness of strategies/activities in everyday life).

Furthermore, of the 427 participants who assessed their overall satisfaction with the program, 71.4% (*n* = 305) were satisfied with the help provided by the program, 85% (*n* = 363) would recommend it to a friend, 76.8% (*n* = 328) would use it again if they needed and 76.1% (*n* = 325) considered they learned relevant information with the program. Additionally, 24.6% (*n* = 105) considered participating in Be a Mom to be too demanding and 10.5% (*n* = 45) considered that the participation was not worth it. Moreover, 90.8% (*n* = 388) of participants rated the quality of Be a Mom as good to excellent. Concerning Be a Mom's access, 91.8% (*n* = 392) of participants accessed the program alone and the remaining 35 mentioned accessing the program with their partner (*n* = 32; 91.4%) or with another family member (*n* = 3; 8.6%).

When comparing high and low-risk groups, a higher proportion of women in the high-risk group considered that participating in Be a Mom was too demanding [$X_{\left(1\right)}^{2}$ = 8.21, *p* = 0.004]. No relationship was found among the groups and the quality of the program [$X_{\left(4\right)}^{2}$ = 3.30, *p* = 0.510] or if they accessed the program alone [$X_{\left(1\right)}^{2}$ = 0.472, *p* = 0.492].

## Discussion

The purpose of the present study was to explore the patterns of usage, reasons for dropout, acceptability and access to the Be a Mom program among postpartum women, and to examine similarities and differences among women presenting high and low risk for PPD. The results of our study highlight that Be a Mom is perceived as an acceptable and useful web-based intervention for both women with high and low risk for PPD. Moreover, the high dropout rates suggest that Be a Mom may be a feasible option for a proportion of women in the postpartum women, although further strategies should be implemented to increase program engagement.

The patterns of usage and acceptability of web-based interventions to prevent and treat PPD in perinatal samples are often overlooked. Despite some evidence on the efficacy of web-based interventions in the postpartum period, literature is scarce and focused mainly on mental health outcomes (e.g., depression and anxiety) in high-risk populations. To the best of our knowledge this is the first study that examined a web-based intervention for this purpose among women presenting high and low risk for PPD.

Considering Be a Mom's feasibility, results have showed that only 27.8% of women have completed the Be a Mom program, 19.4% have partially completed the program and ~63% dropped out. Nevertheless, it is important to note that many of the women who have partially completed the program, even though they did not complete all the modules, still considered Be a Mom useful and the information learned relevant.

The low adherence to the intervention and the high dropout rate are congruent with the results of previous studies with web-based self-guided interventions for PPD (19, 22, 24, 28, 29), which reported rates of adherence between 1 and 37% and dropout rates between 4.5 and 86.9%. The variety of stressors postpartum women have to face in this period such as sleep deprivation, emotional, physical, hormonal and social changes, and the demands of caring for a newborn may have contributed to the low adherence and to women dropping out early from the intervention. In order to increase adherence and lower the dropout rates, the development of intervention programs in which content is divided so that women can access it for a very short period of time during different days can be considered. Moreover, the adoption of engagement strategies (e.g., gamification principles) may increase women's adherence and compliance with the program. In line with this, the introduction of some synchronous support features (with health professionals or with other peers) may also contribute to increased compliance with the program. Additionally, the use of reminders can be intensified. Although, further studies are needed to explore this hypothesis and to test the effectiveness of different approaches to increase women's compliance with the program.

Moreover, when comparing the results of the intervention groups, a higher proportion of women in the low-risk group completed the Be a Mom program and, on average, also completed more modules. Although, both intervention groups considered the Be a Mom program acceptable, relevant and useful, the dropout rate was higher in high-risk women for PPD. A possible explanation for this is that women in the high-risk group, due to their vulnerability factors (e.g., lack of social support), may have more difficulties finding the necessary time and resources to participate in the intervention. Also, and congruently with the dropout rates found, a higher proportion of women in the high-risk group considered participating in Be a Mom to be too demanding. Grounded on these findings, it is important to consider how to adjust the program's length and intensity in future updates of the program, in order to make Be a Mom more adaptable and suitable to the women's needs in this period of high demand. One hypothesis is to conduct further studies to identify the content that is significantly associated with women's improvement in wellbeing, and to make briefer versions of the program. In addition, adapting the program to a mobile-app may be also a way to significantly increase women's access and compliance to the program, since its web-based nature (and computer-based access) may hinder women's access to the platform. On the other hand, considering the self-selection issue of this study, it is possible that women in the low-risk group who enrolled and participated in this study had the perception they could benefit from this intervention or may experience difficulties in this specific period of their life, which could increase their motivation to participate in the intervention. These findings highlight the importance of motivating and generating commitment concerning program's usage among high-risk women, in order to effectively prevent depressive symptoms and promote maternal wellbeing. However, it is important to refer that despite that, previous studies showed the efficacy of Be a Mom in reducing depressive symptoms and in promoting positive mental health even when women did not completed all modules of the program (23, 27).

In line with this, the main reason most women in both intervention groups referred for not completing Be a Mom was lack of time. This could be explained by the demandingness that some postpartum women feel to perform childcare tasks in their transition back to work and the difficulty of reconciling work and family life. When taking together the results of Be a Mom's feasibility and reasons for dropout, it is important to consider that web-based interventions may be a useful and feasible option for some postpartum women, but not for all. Current healthcare services should offer different responses that may be tailored to the specificities of different profiles of women, instead of offering a “one-size-fits-all” approach. Further studies should examine these hypotheses.

The results of Be a mom's acceptability were also globally promising. Most of women were satisfied with the modules (i.e., with the meaning and understanding of the information and usefulness of strategies/activities suggested) and with the help provided by the program. Moreover, the majority of women reported their intention to use the program again if needed, that they would recommend it to a friend and considered the information learned relevant. These results highlight the importance of the Be a Mom intervention for participants. It seems that women learned relevant information and strategies to deal with everyday situations and challenges, helping them to cope with the transition to motherhood, in the recognition of depressive signs/symptoms, and in the improvement of engagement in self-care/family care (baby and one's partner) practices and behaviors.

Furthermore, it is important to mention that women's perceptions of overall satisfaction with the program were congruent with the acceptability results of the previous pilot trial (27). Further studies comparing web-based interventions vs. face-to-face interventions in postpartum women are needed to determine whether online interventions could overcome the barriers of time, motivation, and childcare demands faced by postpartum women.

There are several limitations in the current study that should be noted. First, the online recruitment through social media networks may have produce a self-selection bias. This type of recruitment is most of the times reliant on women's motivation levels and interest to participate in this line of research, which might have some influence in the results. Second, our sample was mostly married or in a relationship and highly educated, which may not be representative of all postpartum women in Portugal. Therefore, the generalizability of the results is consequently limited and future studies should include a more heterogeneous and representative sample. In addition, the women who did not register on Be a Mom had a lower education level than those who registered. Both these issues may highlight that more efforts should be made to make the Be a Mom program accessible to women with higher levels of sociodemographic vulnerability (e.g., low educated single mothers). Finally, our study included only a self-report questionnaire to identify the reasons for dropout and acceptability. Although the reasons to not complete the Be a Mom program have been identified, they should be replicated in other samples using, for example, an interview to clearly establish postpartum women's reasons and perspectives about the program.

In light of what was mentioned, the Be a Mom program could be an addition to supplement interventions for PPD in health centers and public hospitals of the Portuguese National Health Service. Future research should consider expanding the criteria of usability and acceptability of web-based interventions to better reflect the needs of postpartum women.

## Data Availability Statement

The original contributions presented in the study are included in the article/supplementary material, further inquiries can be directed to the corresponding author.

## Ethics Statement

The studies involving human participants were reviewed and approved by Ethics Committee of the Faculty of Psychology and Educational Sciences, University of Coimbra. The patients/participants provided their written informed consent to participate in this study.

## Author Contributions

SX has been responsible for the conceptualization, data collection, data analysis and interpretation, as well as the drafting of the article. FM was responsible for data collection and final revision of the paper. AF designed the study and MC and AF have been responsible for the revision of the article. All authors have reviewed and approved this final version of the manuscript.

## Funding

This project was co-funded by the European Regional Development Fund (FEDER), through the Portugal-2020 Program (PT2020), under the Centre's Regional Operational Program (CENTRO-01-0145-FEDER-028699), and by the Portuguese Foundation for Science and Technology/MCTES through National Funds (PIDDAC). This study was also supported by the Center for Research in Neuropsychology and Cognitive and Behavioral Intervention (CINEICC)—University of Coimbra (UIDB/PSI/00730/2020).

## Conflict of Interest

The authors declare that the research was conducted in the absence of any commercial or financial relationships that could be construed as a potential conflict of interest.

## Publisher's Note

All claims expressed in this article are solely those of the authors and do not necessarily represent those of their affiliated organizations, or those of the publisher, the editors and the reviewers. Any product that may be evaluated in this article, or claim that may be made by its manufacturer, is not guaranteed or endorsed by the publisher.
